# Pulse oximetry values of neonates admitted for care and receiving routine oxygen therapy at a resource‐limited hospital in Kenya

**DOI:** 10.1111/jpc.13742

**Published:** 2017-10-27

**Authors:** Melissa C Morgan, Beth Maina, Mary Waiyego, Catherine Mutinda, Jalemba Aluvaala, Michuki Maina, Mike English

**Affiliations:** ^1^ Department of Paediatrics University of California San Francisco San Francisco California United States; ^2^ Department of Paediatrics Pumwani Maternity Hospital Nairobi Kenya; ^3^ Department of Paediatrics and Child Health University of Nairobi Nairobi Kenya; ^4^ Kenya Medical Research Institute Wellcome Trust Research Programme Nairobi Kenya; ^5^ Nuffield Department of Medicine and Department of Paediatrics University of Oxford Oxford United Kingdom

**Keywords:** Kenya, neonate, oxygen saturation, oxygen therapy, pulse oximetry

## Abstract

**Aim:**

There are 2.7 million neonatal deaths annually, 75% of which occur in sub‐Saharan Africa and South Asia. Effective treatment of hypoxaemia through tailored oxygen therapy could reduce neonatal mortality and prevent oxygen toxicity.

**Methods:**

We undertook a two‐part prospective study of neonates admitted to a neonatal unit in Nairobi, Kenya, between January and December 2015. We determined the prevalence of hypoxaemia and explored associations of clinical risk factors and signs of respiratory distress with hypoxaemia and mortality. After staff training on oxygen saturation (SpO_2_) target ranges, we enrolled a consecutive sample of neonates admitted for oxygen and measured SpO_2_ at 0, 6, 12, 18 and 24 h post‐admission. We estimated the proportion of neonates outside the target range (≥34 weeks: ≥92%; <34 weeks: 89–93%) with 95% confidence intervals (CIs).

**Results:**

A total of 477 neonates were enrolled. Prevalence of hypoxaemia was 29.2%. Retractions (odds ratio (OR) 2.83, 95% CI 1.47–5.47), nasal flaring (OR 2.68, 95% CI 1.51–4.75), and grunting (OR 2.47, 95% CI 1.27–4.80) were significantly associated with hypoxaemia. Nasal flaring (OR 2.85, 95% CI 1.25–6.54), and hypoxaemia (OR 3.06, 95% CI 1.54–6.07) were significantly associated with mortality; 64% of neonates receiving oxygen were out of range at ≥2 time points and 43% at ≥3 time points.

**Conclusion:**

There is a high prevalence of hypoxaemia at admission and a strong association between hypoxaemia and mortality in this Kenyan neonatal unit. Many neonates had out of range SpO_2_ values while receiving oxygen. Further research is needed to test strategies aimed at improving the accuracy of oxygen provision in low‐resource settings.

## What is already known on this topic

1. Out of range oxygen saturation (SpO_2_) levels may complicate neonatal illness and lead to the development of retinopathy of prematurity.
2. Effective monitoring and titration of oxygen therapy could improve neonatal outcomes.
3. Shortages of nurses and pulse oximeters are common in low‐resource facilities.

## What this paper adds

1. In a Kenyan neonatal unit, we found a high prevalence of hypoxaemia at admission and a strong association between hypoxaemia and inpatient mortality.
2. Presence of nasal flaring and hypoxaemia on admission were significantly associated with inpatient mortality.
3. Despite provision of contextually tailored training, many neonates were outside the SpO_2_ target range while receiving oxygen therapy.

Globally, there are 2.7 million neonatal deaths annually.^1^ Preterm birth (<37 weeks gestation) is the leading cause of neonatal death, three‐quarters of which occur in sub‐Saharan Africa and South Asia,^1^ where availability of neonatal intensive care and necessary equipment are limited. Hypoxaemia may complicate neonatal illness and is reported to be common amongst sick neonates admitted to paediatric wards in low‐ and middle‐income countries (LMICs, ranging from 17% to 43%).^2^, ^3^, ^4^, ^5^ In The Gambia, the mortality rate was 75% amongst neonates with hypoxemia, which they defined as oxygen saturation (SpO_2_) <90%.^2^ Effective treatment of hypoxaemia through tailored oxygen therapy could reduce neonatal mortality in low‐resource settings.

Pulse oximetry is the standard of care to non‐invasively monitor SpO_2_ in neonates in developed countries. This is particularly important for preterm neonates, who are at risk for retinopathy of prematurity (ROP).^6^, ^7^, ^8^ Globally, in 2010, an estimated 185 000 preterm infants developed ROP, and ~20 000 became blind or severely visually impaired as a result.^8^ In high‐income countries, ROP is most common in infants born at <26 weeks.^8^ In LMICs, infants with severe ROP have a wider range of birthweights and gestational ages, and rates of disease requiring treatment are higher.^7^ ROP may occur even with non‐invasive oxygen exposure.^9^ Providers thus face a challenge to monitor and titrate oxygen to provide the optimal ‘dose’.^8^, ^10^, ^11^ However, many LMIC facilities do not have sufficient nurses or oximeters to enable continuous monitoring of all neonates receiving oxygen.^7^, ^8^, ^12^, ^13^ Additionally, guidelines and training on pulse oximetry are often inadequate in low‐resource settings.^14^

Several studies have investigated the proportion of time that SpO_2_ was within the target range amongst preterm infants receiving oxygen.^15^, ^16^, ^17^, ^18^, ^19^, ^20^, ^21^, ^22^, ^23^, ^24^, ^25^, ^26^, ^27^ All studies except one were conducted in high‐income settings. These studies showed that, with manual titration, SpO_2_ was outside and/or above the target range 18–84% and 15–58% of time, respectively.^15^, ^16^, ^17^, ^18^, ^19^, ^20^, ^21^, ^22^, ^25^, ^26^ In Colombia, SpO_2_ was within or above the target 34 and 55% of the time, respectively.^21^ Effects of training nurses to target SpO_2_ ranges are varied.^23^, ^27^

This study was conducted at Pumwani Maternity Hospital in Nairobi, Kenya. We aimed (i) to determine the overall prevalence of hypoxaemia in sick neonates admitted to the neonatal unit (Part 1) and (ii) to estimate the proportion of out of range SpO_2_ measurements amongst neonates receiving oxygen therapy (Part 2).

## Methods

### Part 1: Determining prevalence of hypoxaemia

#### Setting

Pumwani Hospital is a maternity facility in Nairobi, located ~1800 m above sea level, which provides care to ~22 000 women and their babies annually. A 60‐bed neonatal unit provides care for neonates requiring medical attention. There are 60–70 neonates routinely being cared for by two to three nurses. Available therapies include oxygen, continuous positive airway pressure (CPAP), intravenous fluids, antibiotics, phototherapy, nasogastric feeds and medications for convulsions and apnoea. Mechanical ventilation and oxygen blenders are not available.

#### Participants

Between January and December 2015, a consecutive sample of ‘sick’ neonates underwent pre‐ and post‐ductal SpO_2_ testing at admission before any oxygen was started. ‘Sick’ neonates were those deemed to require management in the unit (other than observation for low birthweight (LBW, <2.5 kg) and/or maternal illness) by a physician or nurse. Eligible neonates were inborn or transferred to Pumwani during the study period and admitted during normal working hours. Neonates were excluded if transferred for anomalies or severe medical problems within 4 h of birth.

#### Procedures

Measurements were obtained using Lifebox oximeters and neonatal probes, developed for low‐resource settings by the World Health Organization (WHO) and the World Federation of Societies for Anaesthesiologists (Acare Technology Co., New Taipei City, Taiwan).^28^, ^29^, ^30^ Measurements were recorded only when there was a good waveform and SpO_2_ was stable for ≥15 s. Medical officers, who were trained on study objectives and procedures, examined neonates and recorded gestational age, birthweight and clinical signs of respiratory distress on admission. Disposition was obtained from charts at the time of discharge or death. Gestational age was based on last menstrual period (LMP), and Ballard examination was conducted when LMP was unknown or incongruent with appearance (ultrasonography is rarely available). We determined the prevalence of hypoxaemia, calculated as the number of neonates with pre‐ or post‐ductal SpO_2_ below the previously defined local threshold of 89% for *healthy* term and preterm neonates within 24 h at 1800 m,^31^ divided by the total number of neonates admitted during the study period. We determined odds ratios for hypoxaemia and all‐cause inpatient death by gestational age, birthweight and clinical risk factors and signs.

### Part 2: Estimating proportion of SpO_2_ measurements outside target range

#### Participants

Over a 2‐month period, between April and June 2015, a consecutive sample of neonates admitted for oxygen therapy were enrolled. Neonates who were outborn, born outside the study period or required transfer for anomalies or severe medical problems within 4 h of birth were excluded. Charts were tagged to prevent concurrent enrolment in Part 1.

#### Sampling approach

SpO_2_ measurements were obtained at admission and four additional time points post‐admission. To determine sample size, we assumed a worst‐case scenario that 50% of measurements would be out of range. Using a conservative design effect of 2, a sample size of 70 (with 280 post‐admission measurements) provided an effective sample size of 140, which would allow us to estimate a proportion of out of range values of 50% with 95% confidence interval (CI) of 41–59%.

#### Target ranges

Based on the best available evidence to balance risks of ROP and mortality at the time of this study,^32^, ^33^, ^34^ target ranges for term and preterm neonates were established in collaboration with Kenyan paediatricians and nurses, which advised values of SpO_2_ ≥ 92% for neonates ≥34 weeks and SpO_2_ 89–93% for neonates <34 weeks. A training package was provided to staff prior to study implementation, which covered the use of the Lifebox and neonatal probe,^29^, ^30^ target ranges and oxygen management using available resources.

#### Procedures

Using Lifebox oximeters and neonatal probes,^28^ trained nurses obtained SpO_2_ values at 0, 6, 12, 18 and 24 h post‐admission, recording measurements only when there was a good waveform and SpO_2_ was stable for ≥15 s. Due to limited resources (nurses, oximeters), only post‐ductal measurements were obtained. We restricted the number of neonates being concurrently evaluated to 12 to ensure that one oximeter was available to guide therapy for each neonate. When the total number of neonates being actively evaluated reached 12, further enrolment was deferred until a neonate exited this phase. Oxygen was delivered through nasal prongs or masks (for CPAP) from an oxygen cylinder or concentrator. A flow rate of 0.5 L (via nasal prongs) was used to commence therapy, in line with WHO recommendations.^35^ Nurses were advised to decrease oxygen slowly in a stepwise manner, as tolerated, when SpO_2_ was above the target range or stable within the target range for an hour or more. When SpO_2_ was out of range, additional data were prospectively collected, including changes to flow or mode of oxygen delivery, whether oxygen was available and any additional therapies administered. When oxygen was unavailable, reason(s) for lack of availability were recorded, including lack of canisters, masks/prongs or other reason. Gestational age was determined as above. We estimated the proportion of out of range values with 95% CIs. Statistical analyses for both parts of this study were carried out using Stata V.13 (StataCorp, College Station, TX, USA).

### Ethical aspects

All participants received standard care as clinically indicated and available. Following an explanation about the study by a research nurse who spoke the appropriate language, written informed consent was obtained from a parent/guardian. Ethical approval was received from the University of California, San Francisco and the Kenya Medical Research Institute.

## Results

### Part 1

A total of 407 neonates were consecutively enrolled between January and December 2015. All eligible neonates were enrolled. The median gestational age was 39 weeks (10th–90th percentile range 32–40), median chronological age was 1.8 h (10th–90th percentile range 0.2–29.9), median birthweight was 3.0 kg (10th–90th percentile range 1.8–3.6), 54.3% were male, and 61.4% were delivered vaginally. Hypoxaemia (SpO_2_ < 89%) was present in 119 neonates (29.2%, 95% CI: 24.9–33.9). Fifty‐four (13.3%) neonates died during the study period (all‐cause mortality).

Fully adjusted odds ratios (aORs) for hypoxaemia and mortality by clinical risk factors and signs are displayed in Table 1. Retractions (OR 2.83, 95% CI 1.47–5.47), nasal flaring (OR 2.68, 95% CI 1.51–4.75) and grunting (OR 2.47, 95% CI 1.27–4.80) on admission were significantly associated with hypoxaemia. Flaring (OR 2.85, 95% CI 1.25–6.54) and hypoxaemia (OR 3.06, 95% CI 1.54–6.07) on admission were significantly associated with mortality.

**Table 1:** Adjusted odds ratios (AOR) for hypoxaemia and mortality by clinical risk factors and signs of respiratory distress (*n* = 407)

	*n* (%)	Hypoxaemia				Mortality			
		*n* (%)	AOR†	95% CI	*P* value	*n* (%)	AOR‡	95% CI	*P* value
Low birthweight (<2.5 kg)	99 (24.3)	43 (43.4)	0.71	0.30–1.72	0.450	23 (23.2)	1.36	0.45–4.13	0.584
Prematurity (<37 weeks)	87 (21.4)	42 (48.3)	1.44	0.58–3.61	0.432	21 (24.1)	0.69	0.21–2.24	0.534
Retractions	248 (60.9)	103 (41.5)	2.83	1.47–5.47	0.002	49 (19.8)	2.05	0.68–6.16	0.200
Nasal flaring	140 (34.4)	76 (54.3)	2.68	1.51–4.75	0.001	41 (29.3)	2.85	1.25–6.54	0.013
Grunting	87 (21.4)	53 (60.9)	2.47	1.27–4.80	0.008	30 (34.5)	2.23	0.98–5.06	0.055
Hypoxaemia	119 (29.2)					36 (30.3)	3.06	1.54–6.07	0.001

† Adjusted for low birthweight, prematurity, retractions, nasal flaring and grunting.

‡ Adjusted for low birthweight, prematurity, retractions, flaring, grunting and hypoxaemia.

CI, confidence interval.

### Part 2

Seventy neonates were consecutively enrolled between April and June 2015. All eligible neonates were enrolled. The median gestational age was 38 weeks (10th–90th percentile range 30–40), median chronological age was 3.4 h (10th–90th percentile range 0.6–72.6), median birthweight was 2.8 kg (10th–90th percentile range 1.4–3.7), 64.3% were male, and 62.9% were delivered vaginally. Sixty‐four percent of neonates were out of range at ≥2 time points and 43% at ≥3 time points in the first 24 h. Table 2 shows the frequency and proportion of all neonates and preterm and/or LBW neonates by the number of times they were out of range over the first 24 h.

**Table 2:** Frequency of out of range oxygen saturation (SpO_2_) measurements amongst neonates receiving oxygen therapy in the first 24 h of admission (*n* = 70)

Number of times outside target range†	Frequency of all neonates (*n* = 70), *n* (%)	95% CI	Frequency of preterm and/or LBW neonates (*n* = 30), *n* (%)	95% CI
0/5	9 (12.9)	6.1–23.0	2 (6.7)	0.8–22.1
1/5	16 (22.9)	13.7–34.4	5 (16.7)	5.6–34.7
2/5	15 (21.4)	12.5–32.9	7 (23.3)	9.9–42.3
3/5	14 (20.0)	11.4–31.3	7 (23.3)	9.9–42.3
4/5	7 (10.0)	4.1–19.5	7 (23.3)	9.9–42.3
5/5	9 (12.9)	6.1–23.0	2 (6.7)	0.8–22.1

SpO_2_ < 92% for neonates ≥34 weeks gestation.

SpO_2_ < 89% or >93% for neonates <34 weeks gestation.

† Measurements obtained at time of admission and at 6, 12, 18 and 24 h post‐admission.

CI, confidence interval; LBW, low birthweight.

**Figure 1:**
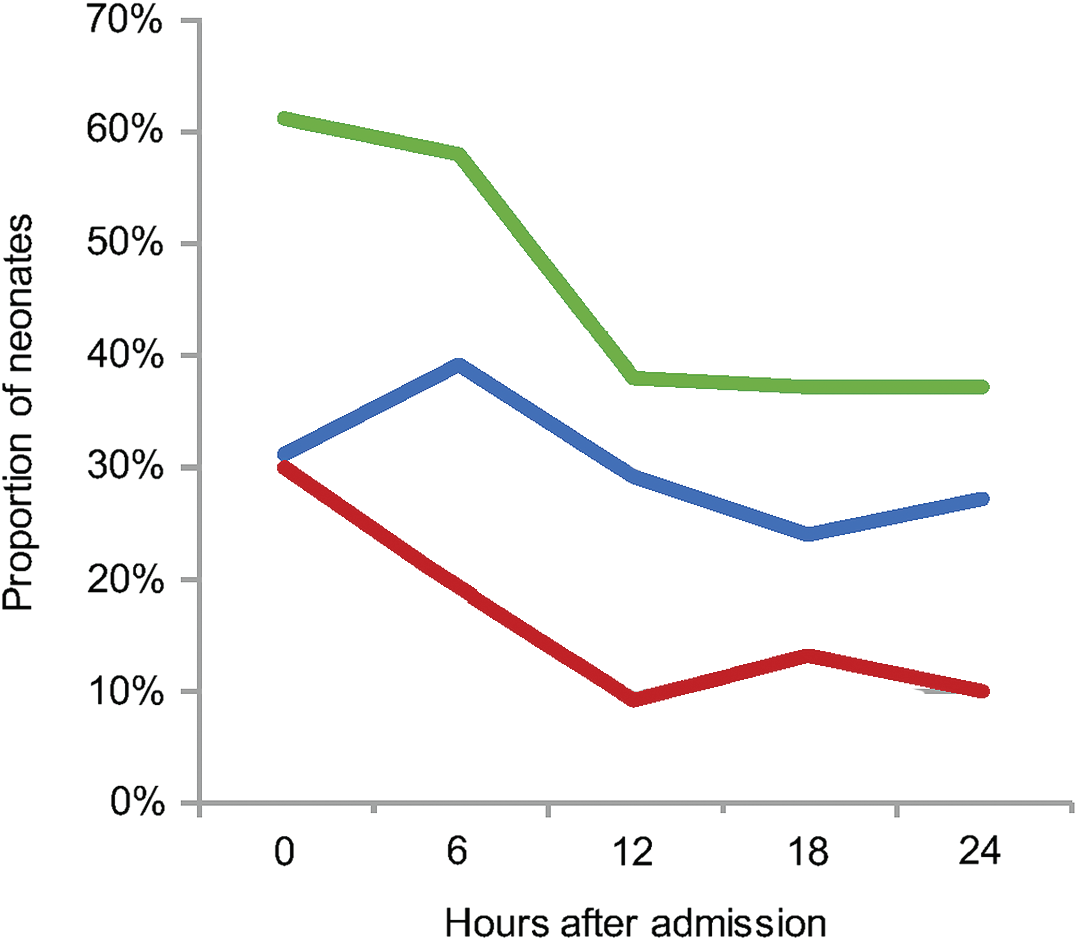
Proportion of neonates outside oxygen saturation target range over the first 24 h of admission. The total proportion of neonates outside the target range decreased from 61% at hour 0 to 58% at hour 6 and 37 to 38% at hours 12, 18 and 24. At hour 0, 31% were above and 30% were below the target range. More neonates were above the target on oxygen therapy from hour 6 onwards. (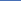), Above target; (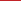), below target; (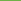), total proportion out of range.

**Figure 2:**
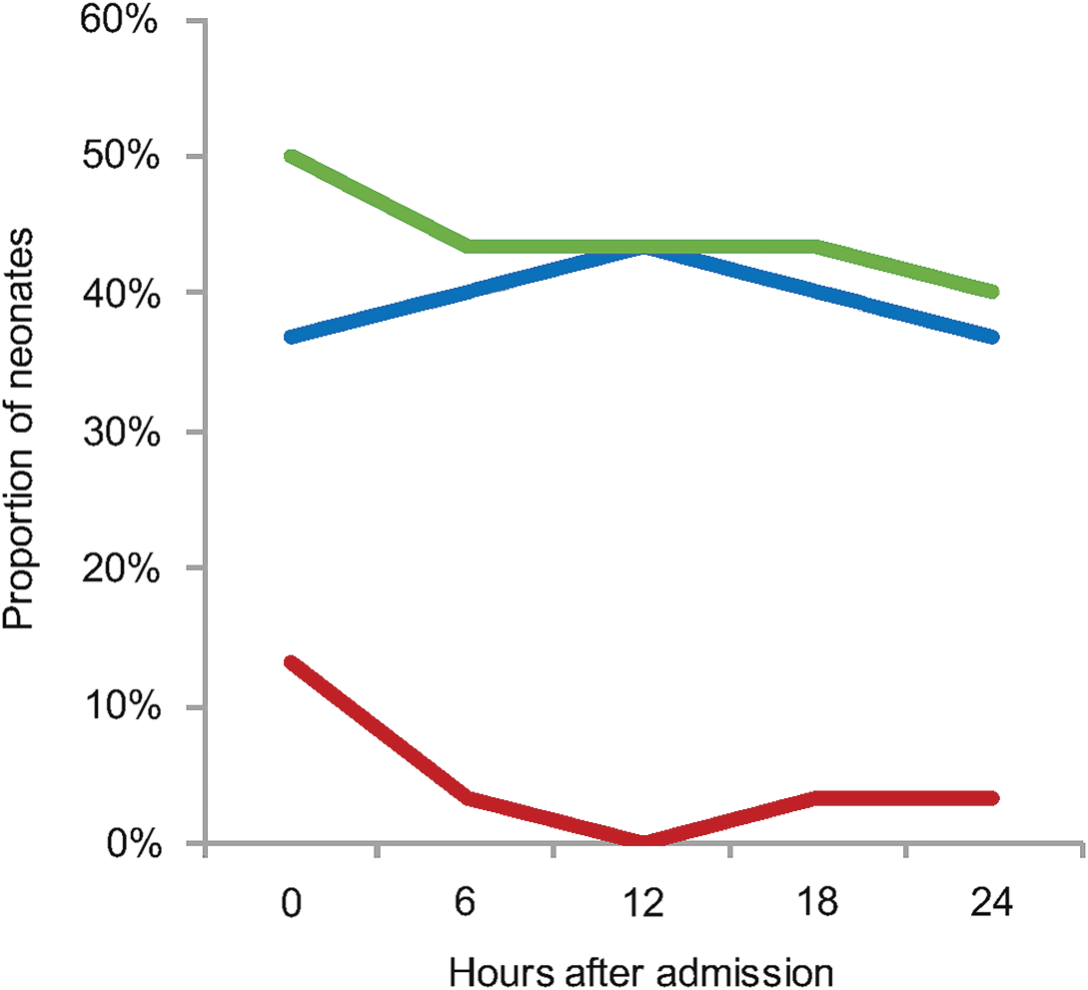
Proportion of preterm and/or low birthweight neonates outside oxygen saturation target range over the first 24 h of admission. The total proportion of preterm and/or low birthweight (LBW) neonates outside the target range decreased from 50% at hour 0 to 43% at hours 6, 12 and 18 and 40% at hour 24. More preterm and/or LBW neonates were above the target on oxygen therapy at all time points, ranging from 37% at hours 0 and 24–40% at hours 6 and 18 and 43% at hour 12. (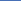), Above target; (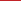), below target; (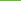), total proportion out of range.

Figure 1 shows the proportion of neonates above and below target, and the total proportion out of range at each time point. Two neonates, both term and >3 kg, remained persistently hypoxaemic (<89% at all time points) despite provision of increasing flow, antibiotics and other therapies. CPAP was not available for either of these neonates. Figure 2 shows the proportion of preterm and/or LBW neonates above and below target and the total proportion out of range. The overall range of SpO_2_ values outside the target range was 33–100%. Only six hyperoxaemic neonates (all preterm) and three hypoxaemic neonates (all term) were ≤3 points outside the target range at a single time point.

When SpO_2_ was below target, flow was increased in 59%, oxygen was restarted in 5%, and CPAP was started in 4%. In 32% of cases, no action was taken. When SpO_2_ was above target, flow was decreased in 25%, and no action was taken in 75%. Oxygen was unavailable twice in the 350 time points assessed. Masks and nasal prongs were available at all time points. Thus, lack of access to basic resources was not a problem; however, availability of CPAP was limited.

## Discussion

This study demonstrates that 29.2% of neonates were hypoxaemic upon admission to a neonatal unit in Nairobi, which predominately cares for newborns delivered in the facility and admitted within 24 h of birth. This is higher than the prevalence of neonatal hypoxaemia reported in Kilifi, Kenya (23%)^3^ and The Gambia (16.5%)^2^ but lower than reported in India (38.5%)^4^ and Papua New Guinea (43%).^5^ Taken together, these data suggest a high burden of neonatal hypoxaemia.

In low‐resource settings, where availability of pulse oximeters may be limited, use of clinical signs to guide decision making is essential. In our study, retractions, nasal flaring or grunting on admission were significantly associated with hypoxaemia. A study in Papua New Guinea also found grunting was significantly associated with hypoxaemia,^5^ while one in Kenya found that flaring or retractions were significantly associated only in univariate analysis.^36^ In our study, flaring was significantly associated with mortality, and adjusted odds of mortality were increased three‐fold in hypoxaemic neonates. This is congruent with studies in The Gambia, Kenya and Papua New Guinea, which found odds or relative risk of death increased by 7.7, 4.3 and 3.1 times, respectively.^2^, ^5^, ^36^ In our study, death occurred despite availability of basic oxygen delivery devices, but with limited availability of CPAP and lack of mechanical ventilation and oxygen blenders.

Periods of hypoxaemia and hyperoxaemia can cause vasoconstriction with subsequent abnormal retinal vascular growth, leading to ROP even in the absence of invasive ventilation.^8^, ^9^ Despite several large randomised trials,^32^, ^33^, ^34^ the optimal SpO_2_ range to reduce risk of ROP, while optimising survival, remains unclear. A recent post‐hoc analysis of data from two trials found that a target range of 85–89%, versus 91–95%, resulted in significantly increased risks of death or disability at 2 years and death alone.^37^ The WHO recommendations state that SpO_2_ should be ≥88% for all newborns in the first hours of life and, additionally, ≤95% for preterm neonates,^35^ similar to the target range for preterm neonates in this study. Beyond the first hours, the WHO recommends that oxygen be commenced for SpO_2_ ≤ 90%. The latter recommendation is supported by the combined trial analysis^37^ and a recent systematic review.^38^

In Part 2, 43% of all neonates and 53% of preterm and/or LBW neonates were out of range at ≥3 time points in the first 24 h. In a trial of 20 non‐invasively ventilated neonates <1000 g in Colombia, the proportion of time within target range was 33.7 ± 4.7% amongst those receiving manual titration.^21^ In our study, 37–43% of preterm and/or LBW neonates were above the target at all time points, higher than one study (25%),^22^ but lower than two others (55–58%).^19^, ^21^ Notably, only one of these studies was conducted in an LMIC. Generalising results from high‐income to LMIC settings is problematic.^39^

We did not consider lack of access to pulse oximeters, oxygen and masks/cannulas major factors during this study, which supplied oximeters. Lack of nursing action in response to the high proportion of out of range values was common. This may reflect high workload and patient‐to‐nurse ratio and the limited range of options available to nurses in this unit. In a high‐income setting, time inside the target range decreased and time above increased with patient‐to‐nurse ratio ≥3.^26^ Other potential causes include inappropriate implementation or insufficient training, related to limited baseline knowledge amongst nurses and sparsity of data in low‐resource settings. Further research is needed to explore strategies aimed at improving oxygen targeting in such settings, where nurses care for many neonates and knowledge is generally low.

This study has limitations. It is possible that prevalence results may have been influenced by training in Part 2. However, this is unlikely as measurements were obtained at admission before any oxygen was started. In Part 1, the 10th percentile of chronological age was 0.2 h (12 min); thus, a small proportion of the hypoxaemic measurements may reflect normal adaptive physiology. Our findings for Part 2 are based on a sample of 70 neonates, among which 30 were preterm and/or LBW, 12 were very preterm (<32 weeks), and 7 were very LBW (<1.5 kg). Our results suggest that oxygen titration may be more challenging in smaller, more preterm neonates; however, our sample is too small to draw any specific conclusions. As ultrasonography is rarely available in this population, we determined gestational age using LMP or Ballard examination. In Bangladesh and Papua New Guinea, mean difference between reference ultrasound and Ballard were −2.8 and 6 days, respectively,^40^, ^41^ and mean difference between ultrasound and LMP was 3 days.^41^ The Lifebox oximeter is not motion‐resistant and has not been validated in neonates. To obtain the most accurate measurements possible, we recorded SpO_2_ only when there was a good waveform and SpO_2_ was stable for ≥15 s. The Lifebox does not enable continuous recording of SpO_2_, which limits direct comparison of our findings to related studies in high‐resource settings. In Part 2, we only measured post‐ductal SpO_2_ due to resource limitations. Studies have demonstrated that pre‐ductal SpO_2_ is higher in the first 4 h^42^, ^43^, ^44^ but becomes equal to post‐ductal SpO_2_ by 18–24 h.^45^, ^46^ Post‐ductal measurements were obtained at all time points, and ≥50% of neonates were above target at all time points; thus, we do not believe this affected our results.

Seven studies reported that use of automated oxygen control devices, which compare incoming SpO_2_ readings with a desired value, increased the proportion of time inside the target range compared to manual titration.^15^, ^16^, ^17^, ^20^, ^21^, ^22^ While promising, such devices are unlikely to be available in low‐resource settings in the near future. Additional strategies for improved oxygen targeting are needed for these settings, where nurses are few and workload is high.^47^, ^48^ Furthermore, wider availability of pulse oximeters in facilities is essential. In 2012, only 4 of 22 Kenyan training hospitals had functional pulse oximeters.^49^ Oxygen provision may be improved through enhanced nurse training, including multiple ‘doses’ over time; increased numbers of neonatal nurses; and training mothers, who often participate in the care of their babies in low‐resource facilities, to titrate oxygen with supervision. A study assessing the effects of a parent training programme for infants being discharged home on oxygen found that those who received training had significantly improved post‐test scores.^49^ Employment of trained care facilitators and development of simple SpO_2_ monitors with algorithm‐based instructions to support mothers could also be beneficial.

## Conclusion

There is a high prevalence of hypoxaemia at admission and a strong association between hypoxaemia and inpatient mortality in this Kenyan neonatal unit, indicating the need for wider deployment of pulse oximeters to LMICs. Despite the provision of a contextually tailored training package, many neonates receiving oxygen were outside the SpO_2_ target range. Further research is needed to test strategies aimed at improving accuracy of oxygen provision in low‐resource settings.
